# Steering on-surface reactions through molecular steric hindrance and molecule-substrate van der Waals interactions

**DOI:** 10.1007/s44214-022-00023-9

**Published:** 2022-12-09

**Authors:** Shiyong Wang, Tomohiko Nishiuchi, Carlo A. Pignedoli, Xuelin Yao, Marco Di Giovannantonio, Yan Zhao, Akimitsu Narita, Xinliang Feng, Klaus Müllen, Pascal Ruffieux, Roman Fasel

**Affiliations:** 1 Empa, Swiss Federal Laboratories for Materials Science and Technology, Überlandstrasse 129, CH-8600 Dübendorf, Switzerland; 2 Key Laboratory of Artificial Structures and Quantum Control (Ministry of Education), Shenyang National Laboratory for Materials Science, School of Physics and Astronomy, Shanghai Jiao Tong University, Shanghai, 200240 China; 3 Max Planck Institute for Polymer Research, Ackermannweg 10, 55128 Mainz, Germany; 4 Department of Chemistry, Graduate School of Science, Osaka University, Suita, 560-0043 Japan; 5 Department of Chemistry and Food Chemistry, Technische Universität Dresden, Mommsenstrasse 4, 01062 Dresden, Germany; 6 Department of Chemistry and Biochemistry, University of Bern, Freiestrasse 3, CH-3012 Bern, Switzerland; 7 Present Address: Istituto di Struttura della Materia—CNR (ISM-CNR), via Fosso del Cavaliere 100, Roma, 00133 Italy

**Keywords:** Graphene nanoribbons, Chemoselectivity, On-surface synthesis, Scanning tunneling spectroscopy, Atomic force microscopy

## Abstract

On-surface synthesis is a rapidly developing field involving chemical reactions on well-defined solid surfaces to access synthesis of low-dimensional organic nanostructures which cannot be achieved via traditional solution chemistry. On-surface reactions critically depend on a high degree of chemoselectivity in order to achieve an optimum balance between target structure and possible side products. Here, we demonstrate synthesis of graphene nanoribbons with a large unit cell based on steric hindrance-induced complete chemoselectivity as revealed by scanning probe microscopy measurements and density functional theory calculations. Our results disclose that combined molecule-substrate van der Waals interactions and intermolecular steric hindrance promote a selective aryl-aryl coupling, giving rise to high-quality uniform graphene nanostructures. The established coupling strategy has been used to synthesize two types of graphene nanoribbons with different edge topologies inducing a pronounced variation of the electronic energy gaps. The demonstrated chemoselectivity is representative for n-anthryl precursor molecules and may be further exploited to synthesize graphene nanoribbons with novel electronic, topological and magnetic properties with implications for electronic and spintronic applications.

**Supplementary Information:**

The online version contains supplementary material available at 10.1007/s44214-022-00023-9.

## Introduction

On-surface synthesis takes advantage of achievements in synthetic chemistry, surface science and materials engineering and—unlike top-down methods—provides access to covalent nanostructures of atomically defined shape and edge structures [1–5]. The typical strategy combines surface-assisted aryl-aryl coupling and subsequent cyclodehydrogenation of a properly designed molecular precursor. In the past decade, this field has rapidly advanced and many intriguing nanostructures ranging from single molecules [6–13], one-dimensional polymers and graphene nanoribbons [2, 5, 14–19] to two-dimensional networks [1, 20, 21] have been realized and characterized down to the atomic level by surface science techniques. The unprecedented structure control enables fine tuning of their electronic, magnetic and topological properties [22–24], with implications for both, fundamental studies as well as potential technological applications. Combined with post-synthesis transfer techniques, electronic devices based on graphene nanoribbons have been achieved, exhibiting high on/off ratio [25], massive Dirac fermion behavior [26] and single electron transistor behavior [27].

On-surface chemoselectivity is the key to realize high-quality target nanostructures with a minimum of possible side products. Many strategies can be used to gain chemoselectivity control to some extent, such as supramolecular templating effects [16, 28–31], substrate templating effects [15, 32–35], kinetic and dynamic effects [36–38], and molecular steric hindrance [39–43]. Although chemoselectivity has been demonstrated in many systems before, it is always desirable to explore new strategies to extend the on-surface synthesis toolbox and design novel nanostructures with tailored properties. Due to strong confinement to two dimensions, molecular steric hindrance plays an important role in on-surface coupling reactions. Previous works demonstrated that molecular steric hindrance can be adopted to realize chemoselectivity. For example, alkyl chain substitution in the ortho position of a para-bisarylalkyne gives rise to a high selectivity toward Glaser coupling due to steric shielding [39], and selective C-H bond activation (cyclo-dehydrogenation) was achieved by weakening the involved C-H bonds by steric hindrance among adjacent hydrogens [41, 44]. In addition, both molecular precursor itself as well as surrounding molecules introduce steric hindrance/shielding, leading the surface coverage an important parameter to control reaction pathways [45].

Here, we demonstrate that molecular steric hindrance can be used to achieve high chemoselectivity towards atomically precise graphene nanoribbons with tailored edge structures. Scanning tunneling microscopy (STM) and non-contact atomic force microscopy (nc-AFM) have been used to resolve the atomic structure of individual precursor monomers, covalent polymers, and GNRs. Our results reveal that molecular hindrance among hydrogen atoms results in highly selective aryl-aryl coupling with well-defined building block orientation, allowing for synthesizing high quality edge-functionalized graphene nanoribbons. Using this coupling concept, two different graphene nanoribbons (GNRs) with different functionalized edges have been fabricated, which exhibit drastically different band gaps as confirmed by combined DFT calculations and scanning tunneling spectroscopy (STS) measurements. Moreover, we further demonstrated the validity of our approach for achieving chemoselectivity by another monomer design with significantly more possible aryl-aryl coupling combinations, confirming the generality of our strategy. Our results provide an efficient way to obtain on-surface chemoselectivity, and can be further extended to fabricate graphene nanostructures with tailored electronic, topological and magnetic properties.

## Steering on-surface reactions through intermolecular steric hindrance

**Figure 1:**
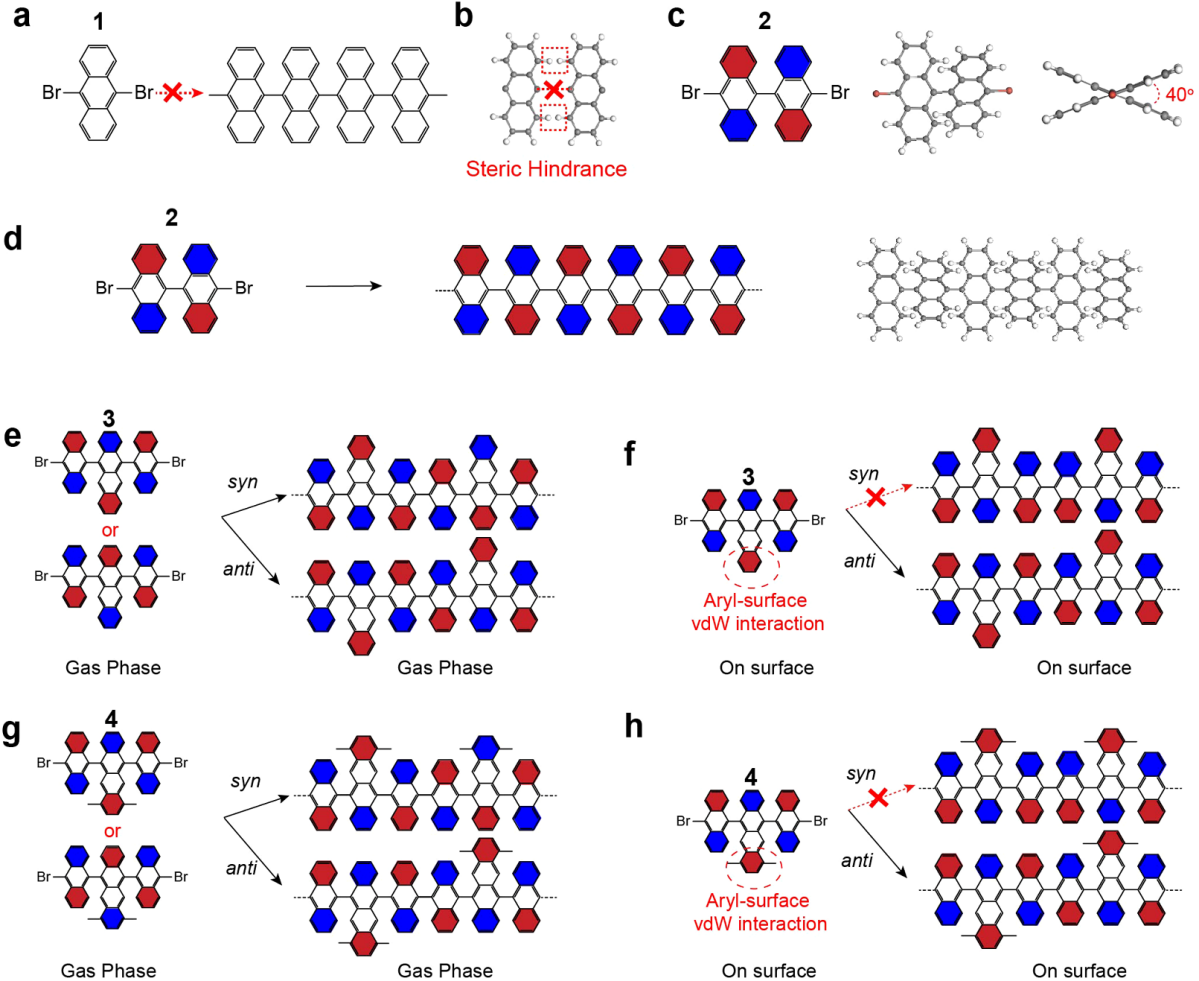
On-surface chemoselectivity mediated by molecular steric hindrance and aryl-surface vdW interactions. (**a**), Prohibited C-C coupling of 9,10-dibromoanthracene precursors. (**b**), ball-and-stick model showing the steric hindrance among opposite hydrogen atoms. Grey, carbon; white, hydrogen. (**c**), Left: the chemical model of precursor 10,10’-dibromo-9,9’-bianthracene with red indicating tilt-down and blue tilt-up. Middle and right: ball-and-stick model showing the alternating tilt of anthracene units. (**d**), linear polymers formed by covalent interlinking of the dehalogenated intermediates. The anthracene units are tilted alternatingly to minimize steric hindrance. (**e**), Left: the chemical model of precursor 5,12-bis(10-bromoanthracen-9-yl)tetracene. Right: the gas-phase reaction scheme towards two different polymers with syn-coupling and anti-coupling. In gas phase, there are two configurations of monomers, which permit both syn- and anti-coupling. (**f**), Left: the chemical model of precursor 5,12-bis(10-bromoanthracen-9-yl)tetracene on surface. Right: the on-surface reaction scheme. On surfaces, there is only one allowed adsorption configurations with the marked aryl group closer to the surface, which permits only the anti-coupling. (**g**)–(**h**), the chemical model of precursor 6,11-bis(10-bromoanthracen-9-yl)-1,4-dimethyltetracene and its reaction scheme in gas phase and on surfaces.

In 2010, we demonstrated the on-surface synthesis of $N=7$ armchair graphene nanoribbons (7-AGNRs) on Au(111) by using the precursor 10,10’-dibromo-9,9’-bianthracene (precursor **2**) [2]. At elevated temperature, the precursor **2** undergoes dehalogenative C-C coupling and subsequent cyclodehydrogenation, resulting in high-quality 7-AGNRs. For comparison, we studied the precursor 9,10-dibromoanthracene (precursor **1**), which has only one anthracene unit and two bromine atoms. Although the precursor **1** shares many similarities with the precursor **2**, the dehalogenative C-C coupling does not work due to steric hindrance. As schematically illustrated in Fig. 1, the precursor **1** adsorbs flat on surface, and steric hindrance among opposing hydrogen atoms prohibits the coupling between monomers. In contrast, Fig. 1(c) shows precursor **2** consists of two anthracene units with a dihedral angle around 40° that is induced by intra-molecular steric hindrance, and thus adopts a non-planar adsorption configuration on Au(111). This non-planarity permits the C-C coupling reaction of adjacent monomers under the condition that approaching anthracene units have an opposite tilt angle. Here, hydrogen atoms are not in the same plane thus removing steric hindrance and allowing precursors to approach enough to allow C-C bond formation between adjacent monomers. Accordingly, anthracene subunits in the growing polymer have a strictly alternating tilt angle (Fig. 1(d)), which demonstrates that molecular hindrance can be effectively exploited to engineer the formation of specific conformational isomers.

In this work, we further expand this concept and demonstrate that molecular hindrance can be utilized to realize coupling chemoselectivity towards the synthesis of GNR with controllably positioned edge functionalizations. We studied the precursor 5,12-bis(10-bromoanthracen-9-yl)tetracene (precursor **3**), which contains one tetracene unit sandwiched by two anthracene units. As illustrated in Fig. 1(e), there are two possible coupling pathways for precursor **3** in gas phase: i) two monomers couple with each other forming a dimer with mirror symmetry (named as syn-coupling), or ii) forming a dimer with center symmetry (named as anti-coupling). For on-surface synthesis, we found that molecular steric hindrance together with molecule-substrate interactions will enable the anti-coupling while completely suppressing the syn-coupling. Upon adsorption on Au(111), the protruding carbon ring of the tetracene unit (highlighted by red) always prefers to adsorb closer to the surface to maximize van der Waals interactions between monomers and the supporting substrate. In this case, intra-molecular steric hindrance will force two side anthracene groups to tilt in the same direction (cf. Fig. 1(f)). Because both terminal anthracene units tilt toward the same direction with respect to the Au(111) surface, the syn-coupling will be completely suppressed due to the large inter-molecular steric hindrance among neighboring hydrogen atoms, while anti-coupling will be allowed by the alternating tilt of anthracene/tetracene units to minimize steric hindrance (cf. Fig. 1(f)). Using similar coupling mechanism, we designed molecular precursor **4** (cf. Figs. 1(g) and 1(h)), which is expected to form uniform graphene nanoribbons hosting alternating armchair and zigzag edges.

## On-surface synthesis of armchair graphene nanoribbon with alternating widths

**Figure 2:**
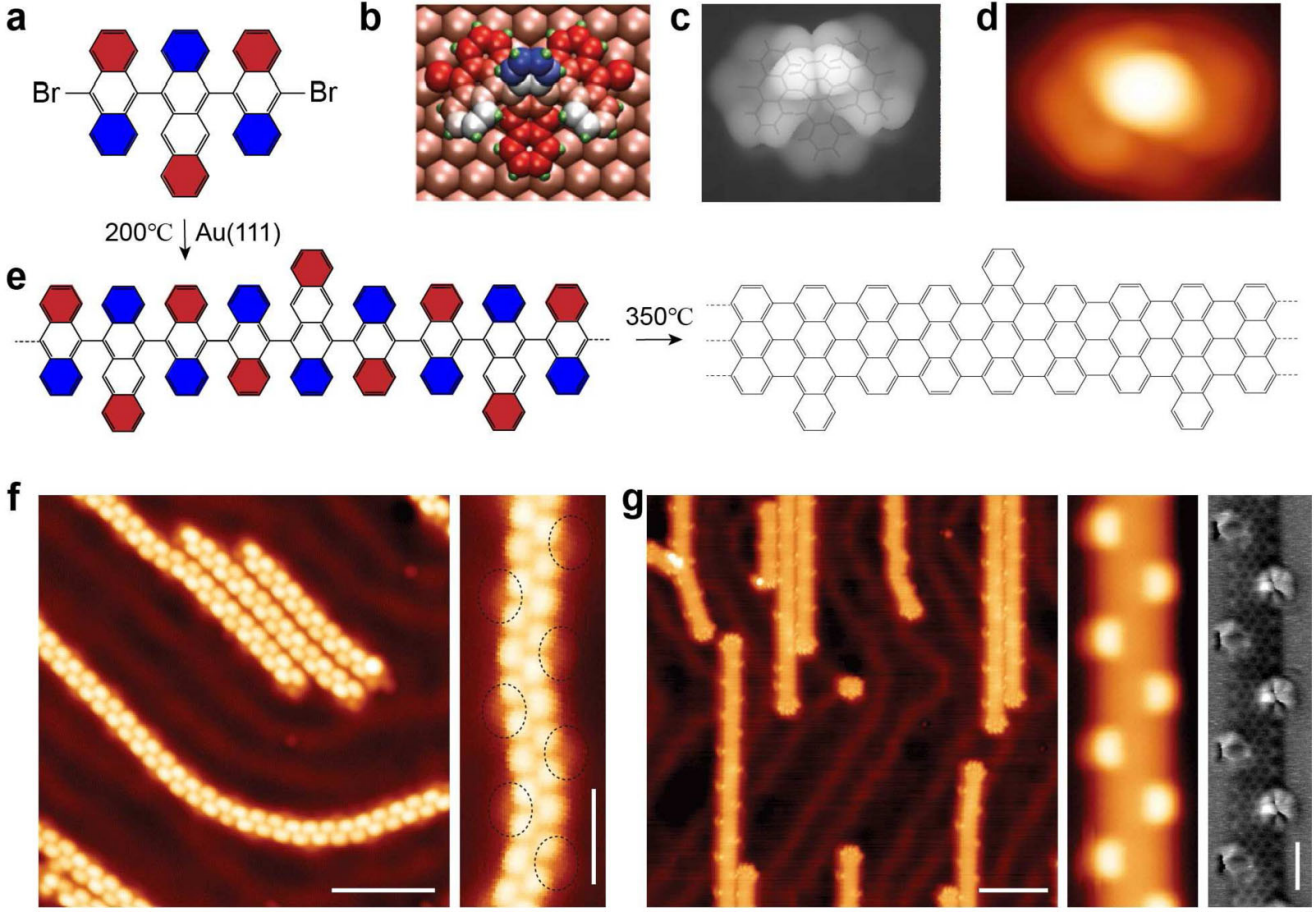
On-surface synthesis of width-modulated graphene nanoribbons. (**a**)–(**d**), adsorption configuration of precursor **3** on Au(111). From left to right: chemical structure, DFT optimized adsorption configuration with blue indicating away from surface and red closer to surface, simulated STM image, and experimental STM image (Bias: 1 V, Current: 50 pA). (**a**), (**e**), Reaction scheme from precursor **3** to polymer intermediates and final graphene nanoribbons, with red indicating closer to surface and blue further away from surface. (**f**), STM image (Bias: 1 V, Current: 50 pA, Scale bar: 5 nm) showing the polymer intermediates. The faint features in the right zoom-in STM image are due to the alternating presence of the protruding carbon ring of tetracene units. (**g**), STM image (Bias: 1 V, Current: 50 pA, Scale bar: 5 nm) showing the resulting GNRs. Zoom-in at the right side show the STM (Bias: 1 V, Current: 50 pA) and nc-AFM image (Oscillation amplitude: 100 pm, Scale bar: 1 nm) of a GNR segment.

To confirm our proposed coupling mechanism, the adsorption configuration of the precursor **3** has been studied by combined density functional theory (DFT) calculations and high-resolution STM imaging. As shown in Fig. 2, DFT calculations reveal the detailed adsorption configuration of precursor **3** on Au(111). The center tetracene unit of all the monomers always tilt in the same direction with respect to the surface plane to maximize van der Waals interactions, which forces the two side anthracene units to tilt toward the same direction. Opposite tilting of the tetracene unit (see Supplementary Fig. 5) is less favorable by 0.3 eV according to our simulations. These adsorption details of the precursor **3** have been confirmed by our low-temperature STM imaging. After deposition of the precursor **3** onto Au(111) held at room temperature, we subsequently cooled the sample to 4 K for imaging. As shown in Fig. 2(c), the STM image of an individual precursor monomer shows a bright protrusion in the center due to the strong bending of the central tetracene unit, which agrees well with the DFT-simulated STM image.

Upon annealing the sample to 200°C, monomers undergo dehalogenative C-C coupling forming intermediate polymers (Fig. 2(f)). The polymers contain alternating protrusions along their long axis with a periodicity of 0.86 nm, which can be elucidated as the successive alternating tilting of anthracene/tetracene units. In addition, the zoom-in STM image resolves the protruding carbon ring of tetracene units, which is imaged as faint features at the polymer edges (marked by dashed dark circles in Fig. 2(f)). The polymers are weakly adsorbed on the Au(111) surface and mostly follow the herringbone reconstruction. Further annealing of the sample to 350°C for 15 minutes induces surface-assisted cyclodehydrogenation of the intermediate polymer and gives rise to atomically precise GNRs with alternating width of 7 and 9 carbon atoms, respectively (named as 7-9-7-AGNR). The small-scale STM image in Fig. 2(g) reveals that 7-9-7-AGNRs host non-planar edges with alternating bright protrusions with a periodicity of 2.6 nm. Non-contact atomic force microscopy (nc-AFM) imaging using a CO-functionalized tungsten tip resolves the chemical structure of a short 7-9-7-AGNR segment, revealing that the protrusions at edges are tilted carbon rings. The out-of-plane tilt of the outer carbon rings is due to steric hindrance between the hydrogen atoms at cove positions. This is confirmed by our DFT simulations where out-of-plane tilting in a three units 7-9-7-AGNR results in an energy gain of 0.5 eV compared to in-plane tilting (see Supplementary Fig. 5) positions.

## On-surface synthesis of graphene nanoribbon with alternating armchair and zigzag edges

**Figure 3:**
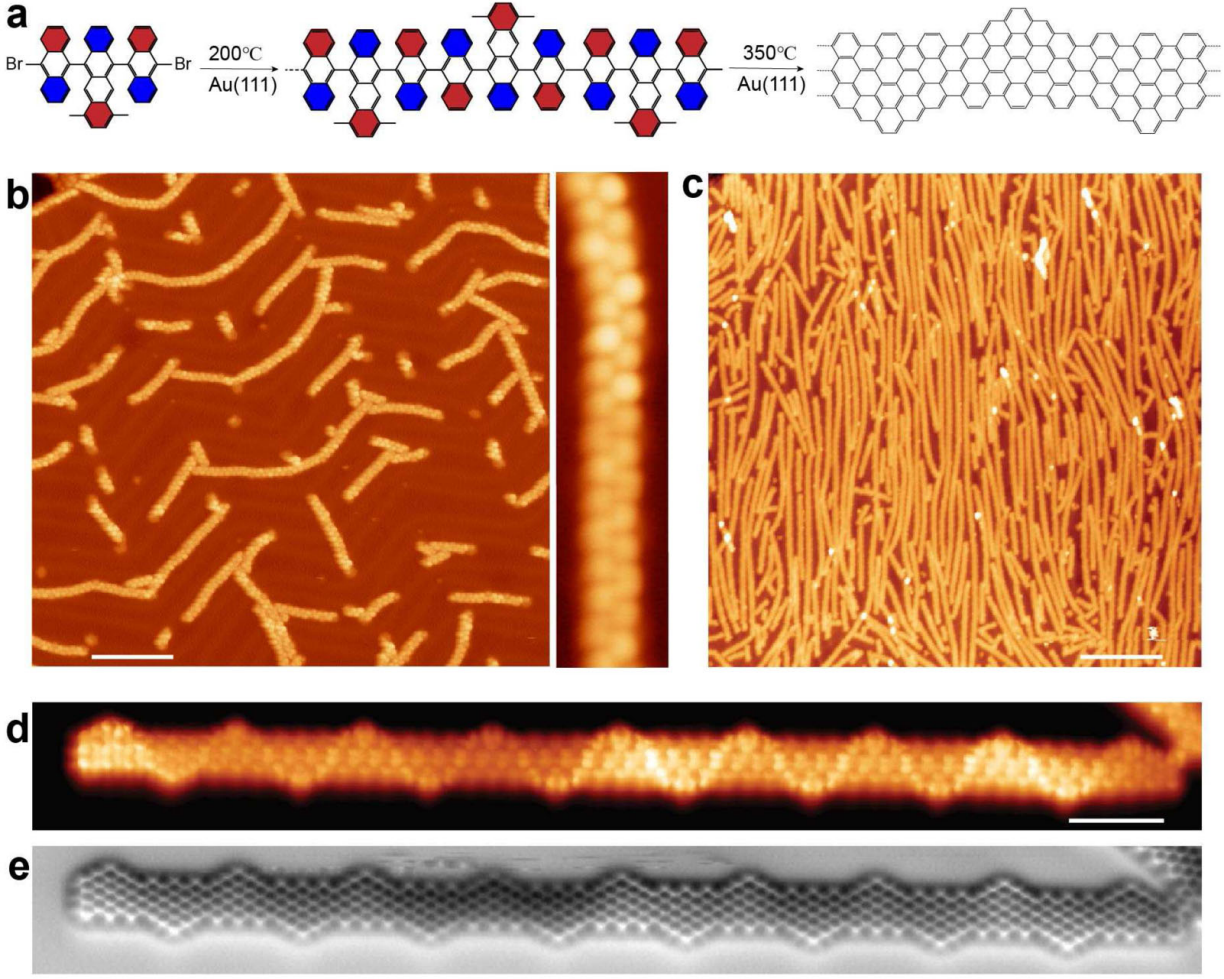
On-surface synthesis of graphene nanoribbons with alternating armchair and zigzag edges. (**a**), Reaction scheme from precursor **4** to polymer intermediates and resulting GNRs, with red indicating closer to surface and blue further away from surface. (**b**), STM image (Bias: 1 V, Current: 50 pA, Scale bar: 10 nm) showing the polymer intermediates. The periodic protrusions in the right zoom-in STM image are due to the alternating tilt of successive anthracene/tetracene units. (**c**), STM image (Bias: 1 V, Current: 50 pA, Scale bar: 15 nm) showing the resulting GNRs. (**d**), (**e**), Simultaneously obtained current image (Bias: 10 mV) and nc-AFM image (Oscillation amplitude: 100 pm, Scale bar: 1 nm) showing a short GNR.

We further demonstrated that our proposed synthesis mechanism can be used to fabricate GNRs with alternating armchair and zigzag edges, which host topological interface states at the zigzag-armchair junctions [23, 46]. As shown in Fig. 3(a), we modified the precursor **3** by adding two methyl groups at the protruding ring of the tetracene subunit (6,11-bis(10-bromoanthracen-9-yl)-1,4-dimethyltetracene, precursor **4**). The precursor **4** shows similar behavior as the precursor **3**. Initial covalent anti-coupling of dehalogenated intermediates at 200°C yields polymer chains (cf. Fig. 3(b)), and cyclodehydrogenation of polymer chains at 350°C gives rise to fully aromatic GNRs with alternating armchair and zigzag edges (7-AGNR+zz). As shown in Fig. 3(c), the resulting high quality GNRs possess an average length of ∼60 nm, suitable for GNR device fabrications. Figures 3(d) and 3(e) display simultaneously acquired current and nc-AFM frequency shift images of a 20 nm long 7-AGNR+zz. The nc-AFM image clearly reveals the chemical structure of 7-AGNR+zz, confirming the high coupling selectivity towards the expected defect-free final GNRs.

## Energy gap of the synthesized graphene nanoribbons

**Figure 4:**
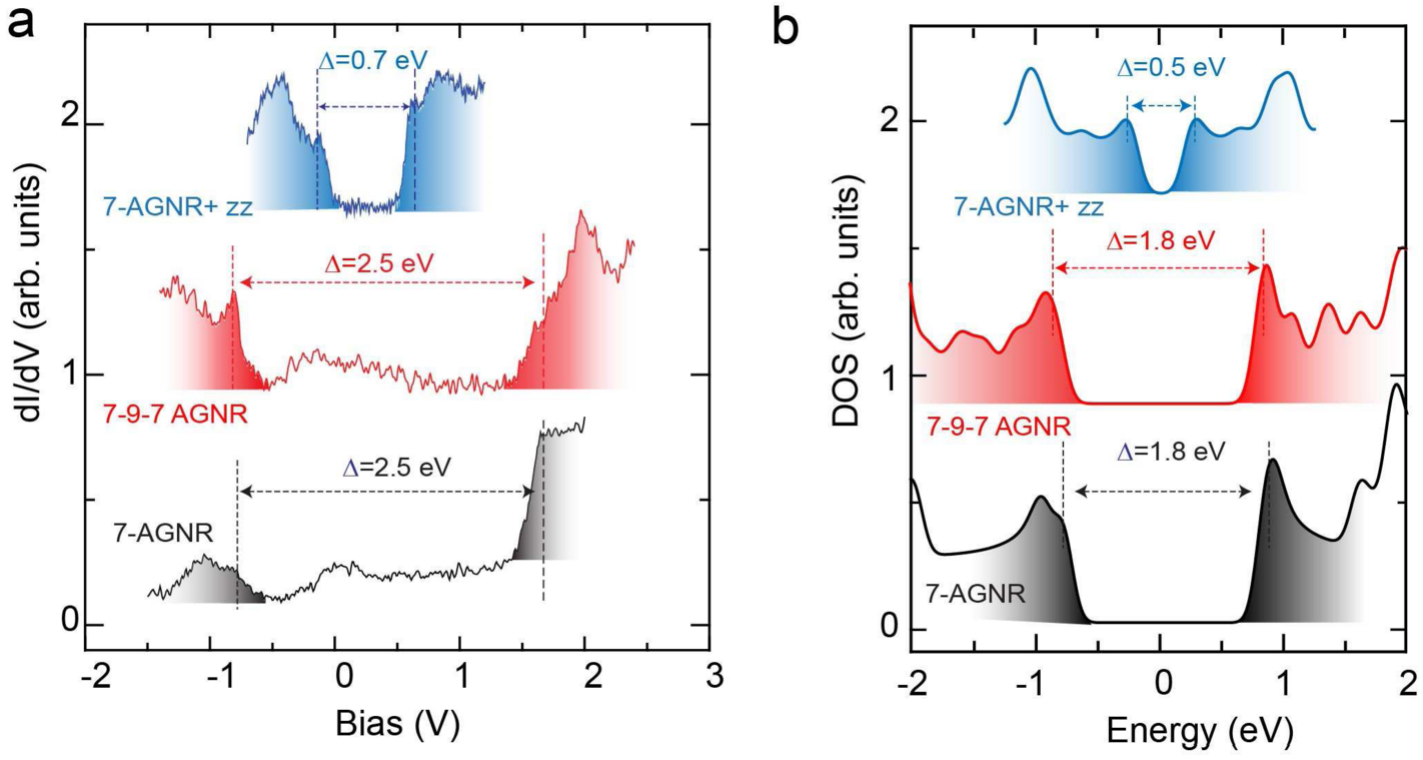
Energy gaps of the synthesized graphene nanoribbons. (**a**), ${dI}/dV$ spectra taken at the edges of the graphene nanoribbons: 7-AGNR (grey), 7-9-7-AGNR (red), and 7-AGNR+zz (blue). (**b**), DFT calculated DOS spectra of these GNRs.

Differential conductance (*dI*/*dV*) spectroscopy has been performed to study the electronic structure of the resulting GNRs. Figure 4(a) shows three ${dI}/dV$ spectra taken at the edges of pristine 7-AGNR, 7-9-7 AGNR, and 7-AGNR+zz, respectively. Both pristine 7-AGNR and edge modified 7-9-7 AGNR show a band gap of 2.5 eV with its valence and conduction band onset at −0.8 eV and 1.7 eV, respectively. Interestingly, a drastically different band gap of 0.7 eV has been observed for 7-AGNR+zz, which is due to the presence of armchair-zigzag interfaces. Each interface provides one localized state, and the coupling of such interface states introduces two additional bands inside the band gap of pristine 7-AGNR [23]. We computed the band structure for infinite 7-9-7 AGNR and 7-AGNR+zz and we compare it to the band structure of 7-AGNR (Fig. 4(b) and Supplementary Fig. 6). The band gap of 1.8 eV for 7-AGNR and 7-9-7 AGNR and of 0.5 eV for 7-AGNR+zz are compatible with the experimental spectroscopy data. For ultra-narrow GNRs, their electronic, magnetic and topological properties depend crucially on edge structures. Recently, our established synthesis method has been further developed to fabricate GNRs hosting flat band and spin chains [47, 48].

## Conclusion and outlook

In conclusion, we demonstrated a strategy to achieve on-surface chemoselectivity by using molecular steric hindrance. By comparing four different molecular precursors, we found that although molecular steric hindrance sometimes prohibits surface coupling reactions, it can be used to obtain full chemoselectivity by suppressing the unwanted C-C reactions. By using our proposed coupling mechanism based on ‘n-anthryl’ precursors, different GNRs with specific patterns of deterministically defined edge extensions have been successfully synthesized and give access to pronounced variation of the electronic band gap, flat bands, topological properties, as well as magnetic properties. Due to strong two-dimensional confinement, molecular steric hindrance effect is generic and we expect it may be further utilized to realize chemoselectivity for synthesizing novel nanostructures, enriching the on-surface synthesis toolbox.

## Methods

A commercial low-temperature STM/AFM (Scienta Omicron) was used for sample preparation and *in situ* characterization under ultra-high vacuum (UHV) conditions (base pressure below $1\times 10^{-10}$ mbar). The Au(111) single crystal was cleaned by standard argon sputtering and annealing cycles. Molecular precursors were dosed into the UHV chamber through a K-cell evaporator. CO molecules were dosed onto the cold sample at around 10 K through a leak value with a pressure of $1\times 10^{-8}$ mbar for 1 minute. STM images were recorded in constant-current mode, and the ${dI}/dV$ spectra were recorded using the lock-in technique ($U_{\mathrm{RMS}}= 20$ mV). nc-AFM images were recorded with a CO-functionalized tip attached to a qPlus tuning fork sensor (resonance frequency $f_{0}=23.5$ kHz).

All DFT simulations where performed with the AiiDAlab platform [49]. The gas phase band structure calculations were performed with the plane wave code quantum espresso [50]. The generalized gradient approximation within the PBE [51] parameterization was used for the exchange correlation functional. Ultrasoft pseudopotentials, from the SSSP library [52] were employed to model the ionic potentials. A cutoff of 50 Ry (400 Ry) was used for the plane wave expansion of the wave functions (charge density). For the convergence of the wave functions a grid of 17 k-points was used to sample the 1D Brillouin zone (BZ). The simulation cell contained 15 Å of vacuum in the non-periodic directions to minimize interactions among periodic replica of the system. The thickness of the vacuum region, the sampling of the BZ and the cutoff ensure convergency of the computed band structures. The atomic positions of the ribbon atoms and the cell dimension along the ribbon axis were optimized till forces were lower than 0.002 eV/A and the pressure in the cell was negligible. The band structures are aligned to the vacuum level computed from the average electrostatic potential in the vacuum region. Calculations for precursors and nanoribbons on the Au(111) substrate were done with the CP2K code [53]. We used simulation cells consisting of four atomic layers of Au along the [111] direction. A layer of hydrogen atoms was used to passivate one side of the slab to suppress the Au(111) surface state. 40 Å of vacuum was included in the simulation cell to decouple the system from its periodic replicas in the direction perpendicular to the surface. The electronic states were expanded with a TZV2P Gaussian basis set [54] for C and H species and a DZVP basis set for Au species. A cutoff of 600 Ry was used for the plane-wave basis set. Norm-conserving Goedecker–Teter–Hutter pseudopotentials [55] were used to represent the frozen core electrons of the atoms. We used the PBE parameterization for the generalized gradient approximation of the exchange-correlation functional [51]. To account for van der Waals interactions, we used the D3 scheme proposed by Grimme [56]. The gold surface was modeled using a supercell of size $50.56 \times 41.21$ Å2 (corresponding to 1088 Au atoms). To obtain the equilibrium geometries, we kept the atomic positions of the bottom two layers of the slab fixed to the ideal bulk positions, and all other atoms were relaxed until forces were lower than 0.005 eV/Å.

## Supplementary Information

Below is the link to the electronic supplementary material.

Supplementary information (PDF 1002 kB)

## Data Availability

Correspondence and requests for materials should be addressed to the corresponding authors.
