# Supplementary material for: Steering on-surface reactions through molecular steric hindrance and molecule-substrate van der Waals interactions
Source: Quantum Front. 2022 Dec 9;1(1):23. doi: 10.1007/s44214-022-00023-9 (PMC9809985; doi:10.1007/s44214-022-00023-9)
Supplement: Supplementary file 1 — Supplementary information (PDF 1002 kB) [file 44214_2022_23_MOESM1_ESM.pdf]

Supplementary Information for

Steering on-surface reactions through molecular steric hindrance and molecule-substrate van der Waals interactions

Shiyong Wang[1,2,¶,], Tomohiko Nishiuchi[3,4,¶], Carlo A. Pignedoli[1,¶], Xuelin, Yao[3], Marco Di Giovannantonio[1,Ψ], Yan Zhao[2], Akimitsu Narita[3], Xinliang Feng[5], Klaus Müllen[3], Pascal Ruffieux[1,*], Roman Fasel[1,6,*]*

[1]*Empa, Swiss Federal Laboratories for Materials Science and Technology, Überlandstrasse 129, CH-8600 Dübendorf, Switzerland.*

[2]*Key Laboratory of Artificial Structures and Quantum Control (Ministry of Education), Shenyang National Laboratory for Materials Science, School of Physics and Astronomy, Shanghai Jiao Tong University, Shanghai 200240, China*

[3]*Max Planck Institute for Polymer Research, Ackermannweg 10, 55128 Mainz, Germany.*

[4]*Department of Chemistry, Graduate School of Science, Osaka University, 560-0043, Japan.*

[5]*Department of Chemistry and Food Chemistry, Technische Universität Dresden, Mommsenstrasse 4, 01062 Dresden, Germany*

[6]*Department of Chemistry and Biochemistry, University of Bern, Freiestrasse 3, CH-3012 Bern, Switzerland*

*Corresponding Authors:*shiyong.wang@sjtu.edu.cn*, *pascal.ruffieux@empa.ch*, roman.fasel@empa.ch*

¶ *Equal contributors*

Ψ Present address: Istituto di Struttura della Materia – CNR (ISM-CNR), via Fosso del Cavaliere 100, Roma 00133, Italy

Detailed synthesis of precursor molecules

All solvents and starting materials were purchased from commercial suppliers, including Aldrich, Acros and Tokyo Chemical Industry, and used without further purification. Column chromatography was performed with silica gel (grain size of 0.047–0.063 mm) and thin layer chromatography was conducted on silica gel coated aluminum sheets with F254 indicator. The ^{1}H NMR and ^{13}C NMR spectra were recorded in solution of CD_2Cl_2 or $C_2D_2Cl_4$ on Bruker Avance 300 MHz spectrometer. Chemical shifts were reported in ppm relative to the residual of solvents (CD_2Cl_2, ^{1}H: 5.32 ppm, ^{13}C: 53.84 ppm; $C_2D_2Cl_4$, ^{1}H: 6.00 ppm, ^{13}C: 73.78 ppm). Coupling constants (*J* values) were recorded in Hertz. Abbreviations: s = singlet, d = doublet, dd = doublet of doublets, m = multiplet). High-resolution mass spectroscopy (HRMS) was performed on a SYNAPT G2 Si high resolution time-of-flight mass spectrometer (Waters Corp., Manchester, UK) by matrix-assisted laser deposition/ionization (MALDI) or electrospray ionization (ESI), calibrated against poly(ethylene glycol). Single crystal diffraction data were collected on a STOE IPDS 2T diffractometer with Mo-K_α Graphite monochromator for all compounds. 10-Dimethyltetracene-5,12-dione (**S4**), 10,10'-dibromo-9,9'-bianthracene (precursor **2**) and 6,11-bis(10-bromoanthracen-9-yl)-1,4-dimethyltetracene (precursor **4**) were synthesized according to our previous reports.[1,2]

Synthesis of precursor 3

5,12-Bis(10-bromoanthracen-9-yl)tetracene (**3**) was synthesized from 5,12-naphthacenequinone (**S2**) by treatment with 10-bromo-9-anthracenyllithium generated by mono-lithiation of 9,10-dibromoanthracene (**S1**) to give dihydroxide **S3**, followed by the dehydroxylation with sodium iodine/sodium hypophosphite monohydrate to give precursor **3** in 68% yield over two steps.

Scheme S1. Synthesis of precursor **3**.

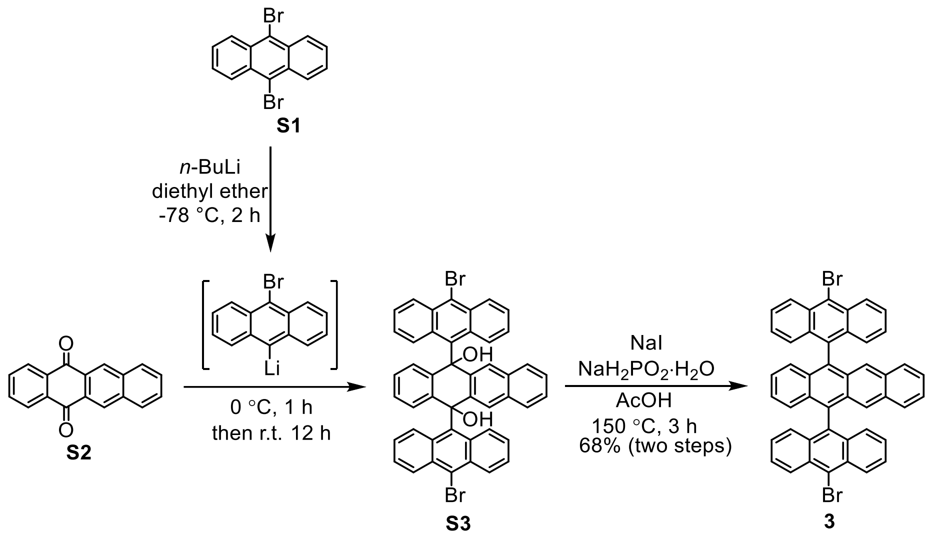

5,12-Bis(10-bromoanthracen-9-yl)tetracene (**3**)

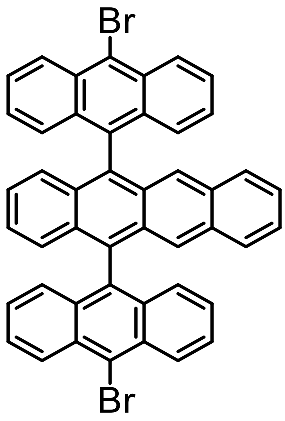

To a suspension of 9,10-dibromoanthracene (**S1**) (400 mg, 1.19 mmol) in dry diethyl ether (40 mL) was added *n*-butyl lithium (*n*-BuLi, 1.6 M in hexanes) (0.60 mL, 0.96 mmol) dropwise at −78 °C. The reaction mixture was stirred at −78 °C for 1 h under argon to obtain an orange solution of 10-bromo-9-anthracenyllithium. To a suspension of 5,12-naphthacenequinone (**S2**) (92.2 mg, 0.357 mmol) in dry diethyl ether (10 mL) was added at 0 °C the separately prepared lithium reagent via a double-tipped needle under argon. The reaction mixture was stirred at 0 °C for 1 h, and then allowed to gradually warm to room temperature. After stirring for 12 h, the reaction was quenched by adding 5 mL of glacial acetic acid. The precipitates were collected by filtration and washed with diethyl ether. To the resulting crude material (**S3**) placed in 100-mL round-bottom flask was added glacial acetic acid (45 mL), NaI (523 mg, 3.50 mmol) and $NaH_2PO_2{\cdot}H_2O$ (568 mg, 5.36 mmol). The reaction mixture was heated at 150 °C for 6 h under the exclusion of light. After cooling down to room temperature, the precipitations were

collected by filtration, and washed with water and methanol. The crude product was then recrystallized through slow diffusion of degassed methanol into its degassed solution in tetrahydrofuran under argon to afford precursor **3** (179 mg, 68% yield) as yellowish-orange solid. ^{1}H NMR (300 MHz, $C_2D_2Cl_4$, 298 K, ppm) *δ* 8.81 (d, *J* = 8.9 Hz, 4H), 7.90 (s, 2H), 7.76 – 7.74 (m, 5H), 7.55 – 7.47 (m, 2H), 7.41 – 7.28 (m, 9H), 7.22 – 7.14 (m, 4H), 7.10 – 7.06 (m, 2H); ^{13}C NMR (75 MHz, $C_2D_2Cl_4$, 298 K, ppm) *δ* 133.98, 133.51, 132.36, 131.19, 130.81, 130.46, 129.91, 128.11, 127.98, 127.41, 127.32, 126.94, 126.48, 125.91, 125.75, 125.49, 123.77. HRMS (MALDI-TOF, positive) *m/z*: Calcd for $C_{46}H_{26}Br_2$: 736.0401; Found: 736.0388 $[M]^+$.

Single crystal X-ray diffraction analysis

Single crystal of precursor **3** suitable for X-ray analysis was obtained by slow diffusion of degassed methanol into a degassed solution of **3** in tetrahydrofuran under argon and exclusion of light. The structure was deposited at Cambridge Data Centre (CCDC number: 2217018).

Crystal data

formula	$C_{46}H_{26}Br_2$	
molecular weight	738.52 $gmol^{-1}$	
absorption	μ = 1.968 mm^{-1} correction with 6 crystal faces	
transmission	T_{min} = 0.55846, T_{max} = 0.8855	
crystal size	0.06 x 0.12 x 0.33 mm^3 yellow needle	
space group	P -1 (triclinic)	
lattice parameters	a = 8.9308(6)Å	α =76.682(5)°
(calculate from	b = 12.4018(7)Å	ß = 86.554(5)°
12775 reflections with	c = 20.0180(13)Å	γ = 77.877(5)°
2.5° < θ < 28.2°)	V = 2109.3(2)$Å^3$	z = 2 F(000) =
944		
temperature	−80°C	
density	d_{xray} = 1.447 gcm^{-3}	

Data collection

diffractometer	STOE IPDS 2T
radiation	Mo-K_α Graphite monochromator
Scan – type	ω scans
Scan – width	1°
scan range	2° ≤ θ < 28°

	$-11 \leq h \leq 11$ $-16 \leq k \leq 16$ $-26 \leq l \leq 26$
number of reflections:	
measured	21885
unique	10030 (R_{int} = 0.0359)
observed	5006 ($\lvert F\rvert/\sigma(F) > 4.0$)

Data correction, structure solution and refinement

corrections	Lorentz and polarisation correction.
Structure solution	Program: SHELXT-2014
refinement	Program: SHELXL-2018 (full matrix). 541 refined parameters, weighting scheme: $w=1/[\sigma^2(F_o^2) + (0.0918*P)^2+2.35*P]$ with $(Max(F_o^2,0)+2*F_c^2)/3$. H-atoms at calculated positions and refined with isotropic displacement parameters, non H-atoms refined anisotropically.
R-values	wR2 = 0.2093 (R1 = 0.0670 for observed reflections, 0.1429 for all reflections)
goodness of fit	S = 1.014
maximum deviation of parameters	0.001 * e.s.d
maximum peak height in diff. Fourier synthesis	1.07, −0.98 eÅ^{-3} near Bromine
remark	crystal contains 2.5mol THF / molecule, one of the THF's is highly disordered and could not be refined – SQUEEZE was used.

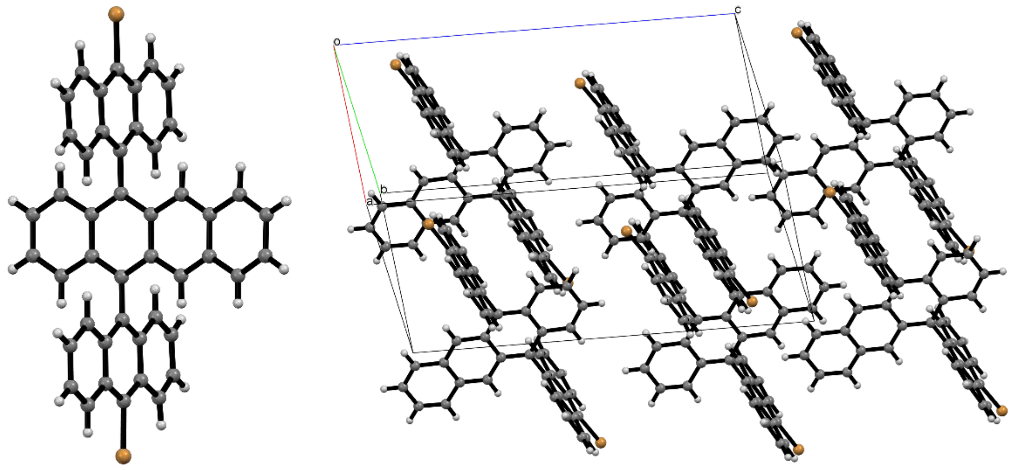

Supplementary Figure S1. X-ray single-crystal structure of precursor **3** (ORTEP drawings with thermal ellipsoids at 50% probability, solvent molecules were removed for clarity).

NMR spectroscopy and mass spectrometry

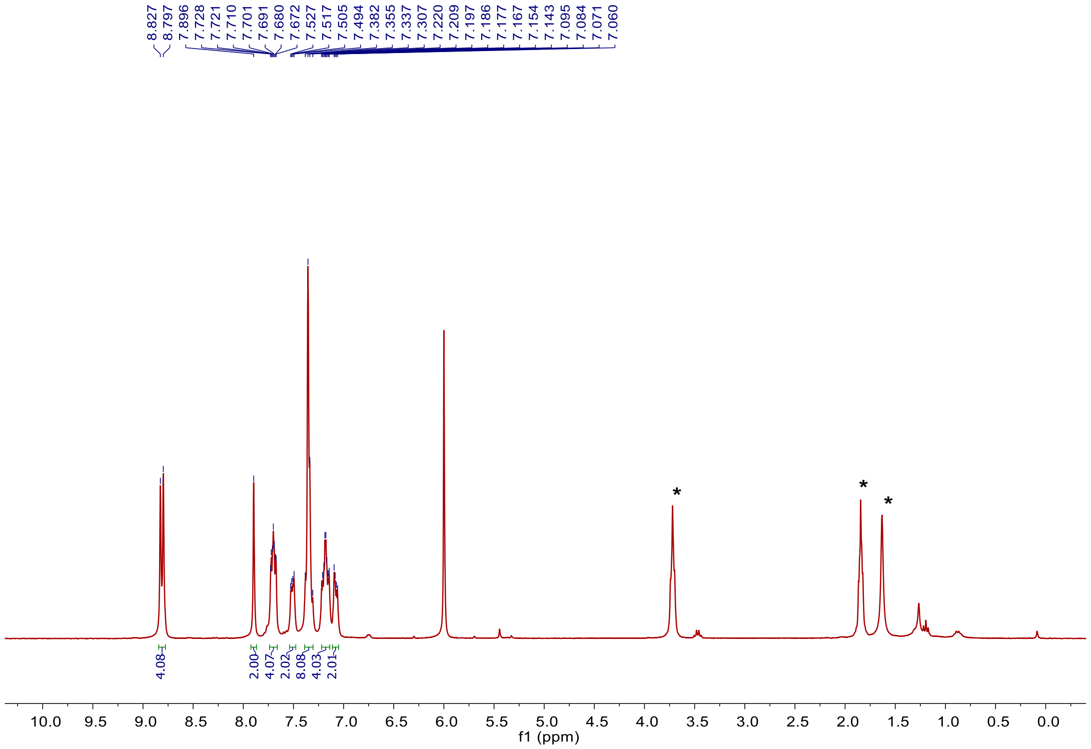

Supplementary Figure S2. ^{1}H NMR spectrum of precursor **3** (300 MHz, $C_2D_2Cl_4$, 298 K). The asterisks indicate signals from THF solvent and water residues.

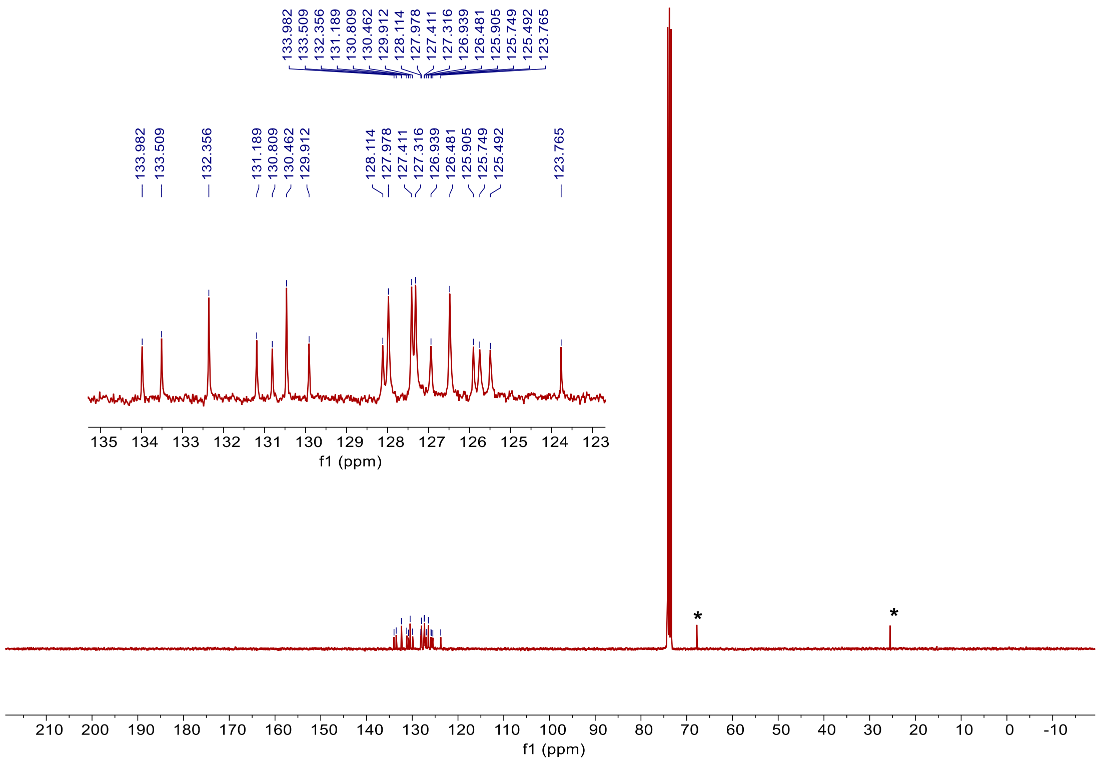

Supplementary Figure S3. ^{13}C NMR spectrum of precursor **3** (75 MHz, $C_2D_2Cl_4$, 298 K). The asterisks indicate signals from THF solvent.

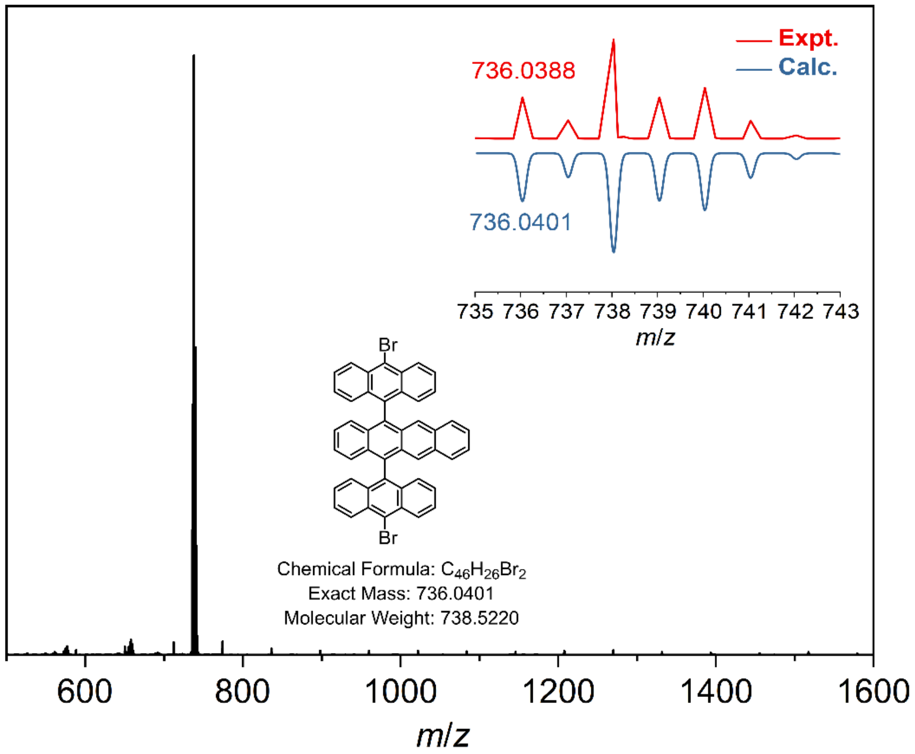

Supplementary Figure S4. High-resolution MALDI-TOF MS spectrum of precursor **3**. Inset displays the isotopic distribution in comparison with the simulated pattern.

DFT-calculated equilibrium adsorption geometries

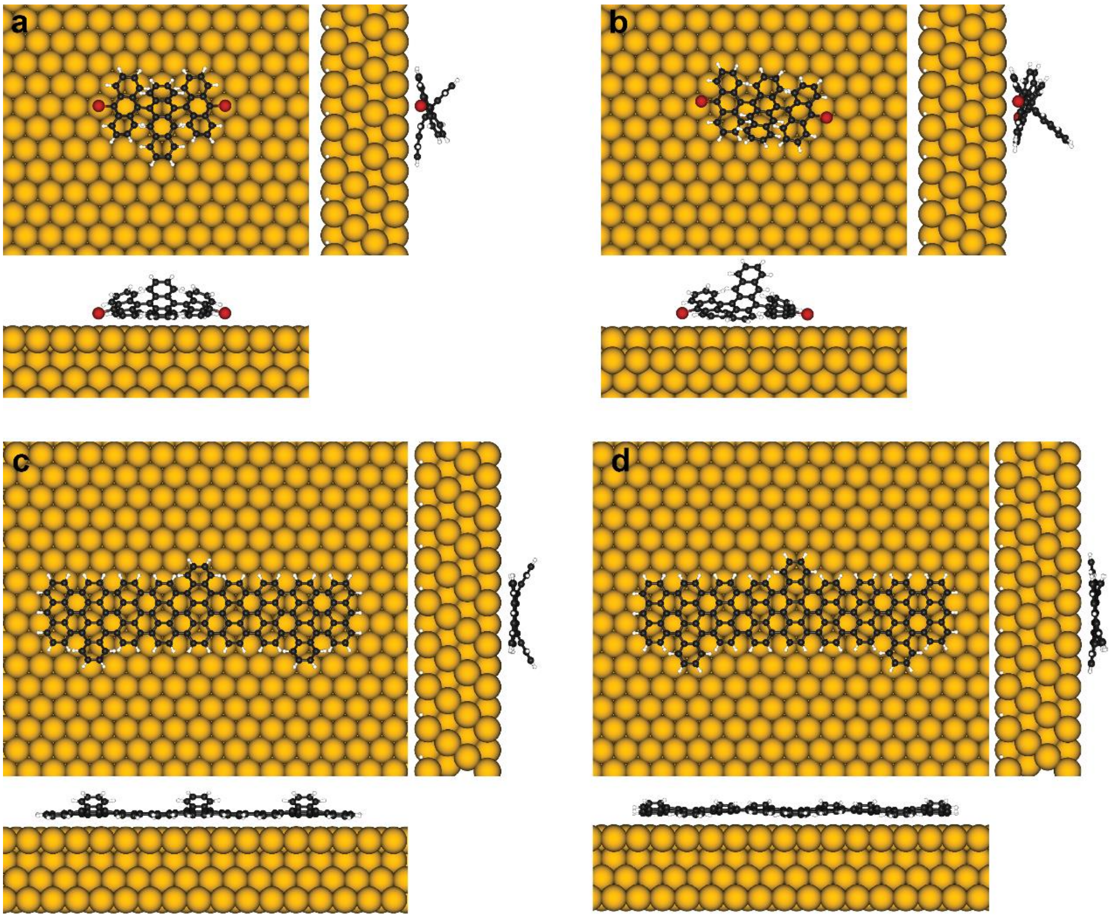

Supplementary Figure S5. DFT-calculated equilibrium adsorption geometries. a, Lowest energy conformation for precursor 3 on the substrate. Upwards tilting (b) of the tetracene unit results in an energy cost of 0.3 eV. c, Lowest energy adsorption geometry for a finite 7-9-7 AGNR composed of three precursor units. In-plane tilting (d) of the outer phenyl rings results in an energy loss of 0.5 eV. Yellow, red, black, and white spheres represent respectively Au, Br, C, and H atoms.

Band structure and density of states of edge-extended graphene nanoribbons

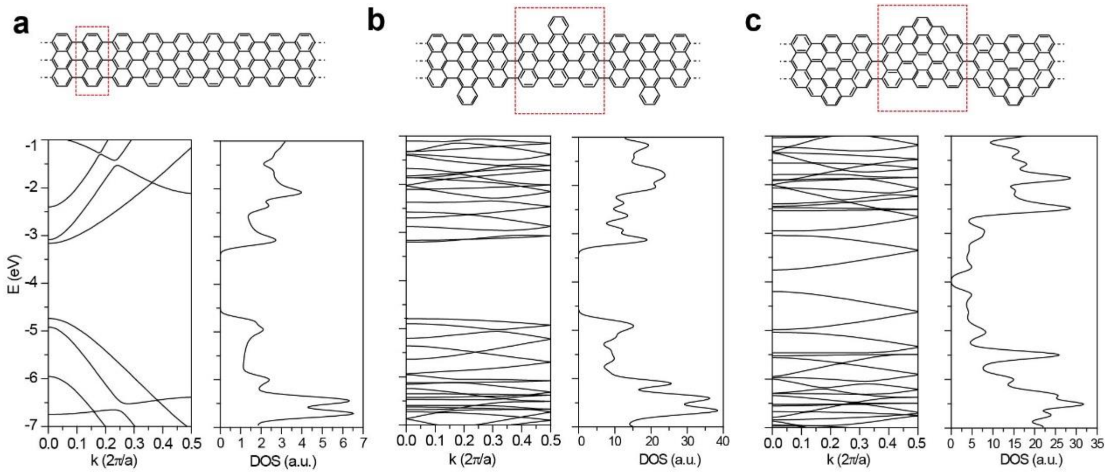

Supplementary Figure S6. Band structure and density of states of the synthesized graphene nanoribbons. The dashed boxes indicate the unit cell used in the gas phase DFT calculations. Periodic boundary conditions are applied. The band gap of 7-AGNR-zz (c) is significantly smaller than 7-AGNR (a) and 7-9-7 AGNR (b) due to the presence of two additional bands originating from the coupling of zigzag interface states.
